# A case series of primary urinary tract neuroendocrine neoplasms

**DOI:** 10.3389/fonc.2026.1816850

**Published:** 2026-05-04

**Authors:** Yang Zheng, Wenfu Wang, Yongheng Zhou, Meikai Zhu, Zhiwen Jiang, Yong Wang, Benkang Shi, Yaofeng Zhu

**Affiliations:** 1 Department of Urology, Qilu hospital of Shandong University, Jinan, Shandong, China; 2 Department of Urology, Reproductive Medicine Center of Jinan Maternity and Child Care Hospital Affiliated to Shandong First Medical University, Jinan, China

**Keywords:** primary urinary cancer, neuroendocrine neoplasms, rare disease, case report, short review

## Abstract

**Background:**

Neuroendocrine neoplasms (NENs) are a heterogeneous group of tumors that originate from neuroendocrine cells. They are common in the digestive system (such as the stomach, intestines, and pancreas) and the respiratory system, but they are rare in the urinary system, comprising less than 1%–2% of urinary malignancies. These tumors typically lack specific clinical symptoms and are usually diagnosed through postoperative pathological examinations. Due to their rarity, the clinical awareness of NENs remains insufficient in the community.

**Methods:**

Clinical data of 10 patients diagnosed with urinary tract neuroendocrine neoplasms (UT-NENs) were retrospectively collected and analyzed. We summarized their clinical characteristics and treatment outcomes, with the aim to improve our understanding of urinary tract NENs and further optimize personalized therapeutic strategies for these rare tumors.

**Result:**

In our study, 4 out of 10 patients were admitted for hematuria. Seven out of 10 patients had lesions in the bladder. Five of 6 cases with NEN muscularis propria invasion occurred in the bladder. All 3 deceased patients had bladder NEN. None of them received postoperative radiotherapy and chemotherapy. Five out of 6 surviving patients received postoperative radiotherapy and chemotherapy.

**Conclusion:**

Hematuria is the main symptom in UT-NENs. The bladder is the most common location of UT-NENs, while bladder lesions are more likely to present as poorly-differentiated neuroendocrine carcinoma. In the present case series, patients who received postoperative radiotherapy and chemotherapy showed a trend toward better long-term prognosis and survival.

## 1 Introduction

Neuroendocrine neoplasms (NENs) are a heterogeneous group of tumors that originate from neuroendocrine cells (1). NENs are common in the digestive system and the respiratory system, but they are rare in the urinary system, comprising less than 1%–2% of urinary malignancies (2). These tumors typically lack specific clinical symptoms leading to considerable difficulty in preoperative diagnosis, missed diagnosis, and delayed clinical intervention. Effective preoperative diagnostic strategies for early detection of urinary system neuroendocrine neoplasms are lacking, and clinical awareness of these tumors remains insufficient in the clinical community. Accordingly, this study was conducted to enrich clinical data and enhance the recognition of this rare neoplasm among clinicians.

## 2 Research subjects and methods

### 2.1 Research subjects

**Figure 1:**
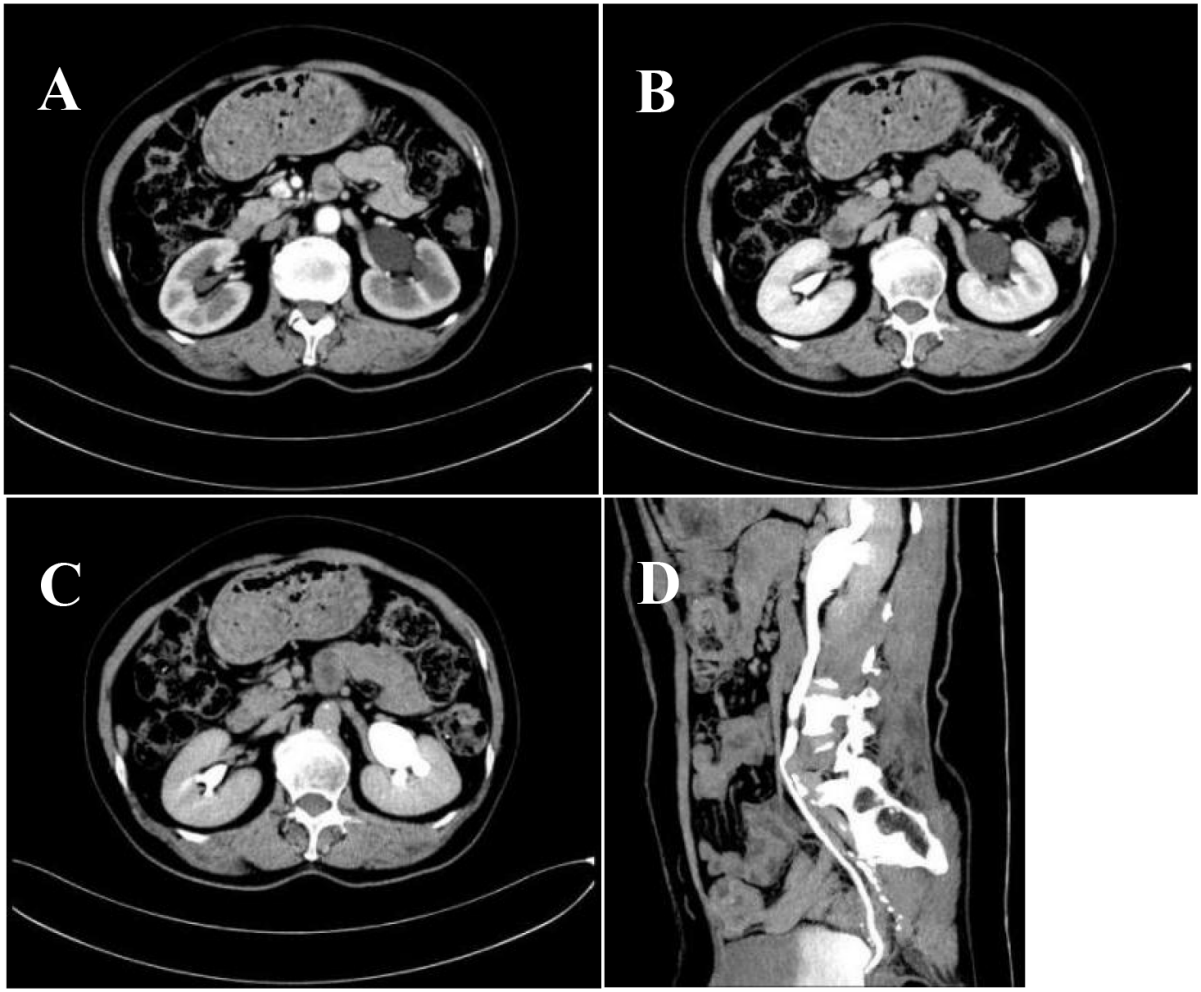
Contrast-Enhanced CT: **(A)** Arterial phase; **(B)** Venous phase; **(C)** Excretory phase; **(D)**. The middle segment of the left ureter is not visible in the coronal position.

**Figure 2:**
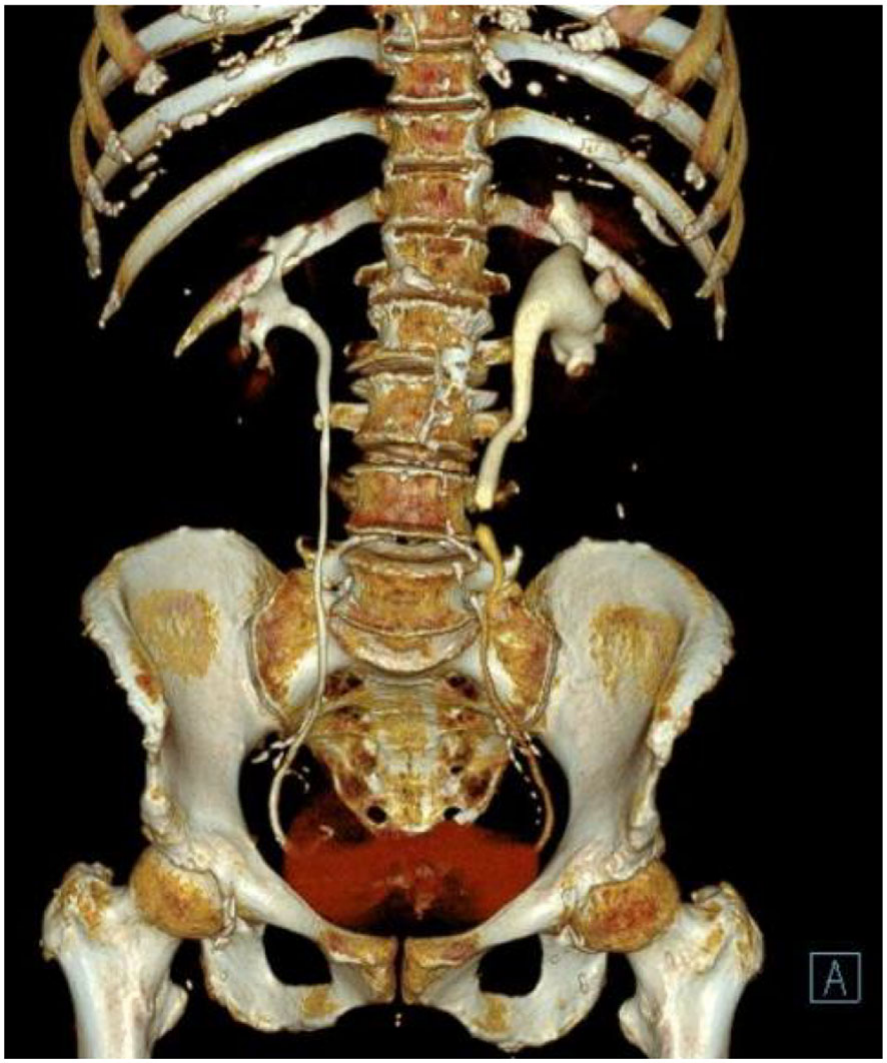
3D reconstruction of the urinary tract: a localized stenosis in the middle segment of the ureter with pyeloureterectasis and hydronephrosis.

We collected 10 patients’ information with urinary tract neuroendocrine neoplasms (UT−NENs) in the Department of Urology, Qilu Hospital, Shandong University from February 2019 to June 2024. The information includes age, gender, presenting symptoms, lesion location, medical history, imaging examinations such as contrast-enhanced CT (CE-CT) and CTU, and biopsy or surgical pathology. All patients were aged between 40 and 85 years. **Figure 1**, **2**, **3** show the contrast-enhanced CT image, 3D reconstruction of the urinary tract and pathological image of a UT−NEN patient.

**Figure 3:**
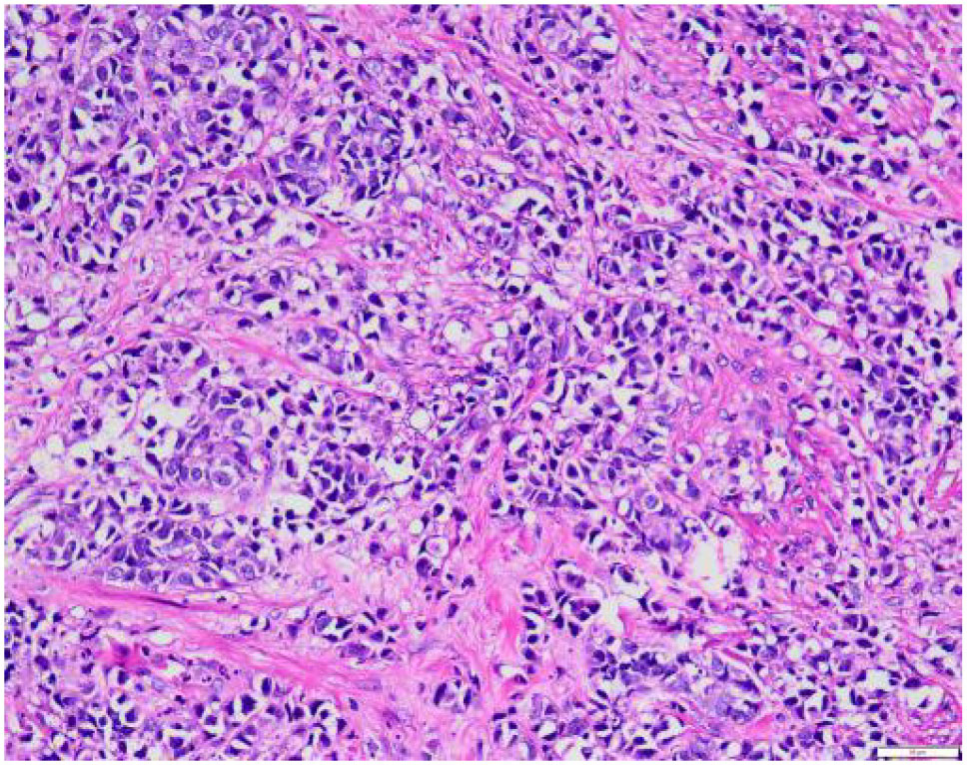
Hematoxylin-EosinStaining Histopathological section (HE×200). The tumor contains various atypical cells and mototic figure.

### 2.2 Methods

This was a retrospective, single-center case series report.The study involving human participants was reviewed and approved by the Ethics Committee of Qilu Hospital of Shandong University. Written informed consent was obtained from all patients, and all patient information was anonymized and de-identified to protect privacy. All procedures followed national ethical standards and the 1964 Declaration of Helsinki. We collected 8 representative clinical parameters from 10 patients with UT-NENs in the Department of Urology, Qilu Hospital, Shandong University, between February 2019 and June 2024, including age, gender, main symptoms, lesion location, surgical treatment, pathological type, muscularis propria invasion, and clinical outcome. The diagnoses of all patients were histopathologically confirmed based on surgical or biopsy specimen examination according to the 2016 WHO pathological diagnostic criteria (3). Hematoxylin-eosin (HE) staining and immunohistochemical analysis were performed according to standard laboratory procedures. By the end of June 2025, all 10 patients had been followed up by telephone. The follow-up duration ranged from 2 to 62 months, with a median follow-up of 8.5 months. The variation in follow-up time was due to the retrospective nature and rarity of the disease. Clinical characteristics, treatment patterns, and follow-up outcomes were summarized. All data were descriptively analyzed without inferential statistics.

## 3 Results

### 3.1 Main symptoms

**Table 1** presents the clinical data of the 10 patients. In our study, 4 patients were admitted due to hematuria, 2 were admitted for pathological findings after cystotomy, 2 for hydronephrosis, and 2 for dysuria and painful urination. Seven patients had lesions located in the bladder, and 3 had lesions in the ureter.

**Table 1:** Data of patients with urinary system NENs.

Patient number	Age/Y	Gender	Main symptom	Lesion location	Operation	Pathology/cell type	Muscle infiltration	Outcome	Follow-up duration/mo
1	84	female	hematuria	kidney/ureter	Nephroureterectomy+Partial cystectomy	Mixed	No	Lost	4
2	73	male	hematuria	bladder	Bladder resection	Mixed	Yes	Survival	10
3	69	male	hematuria/dysuria/painful urination	bladder	Bladder resection	Large	No description	Survival	10
4	71	male	pathology/no symptom	bladder	Partial cystectomy	Large	No description	Dead	58
5	44	male	hematuria	bladder	Radical cystectomy	Mixed	Yes	Survival	9
6	68	male	pathology/no symptom	bladder	Radical cystectomy	None	Yes	Dead	59
7	44	male	space occupy/hydronephrosis	kidney/ureter	Nephroureterectomy	None	Yes	Survival	4
8	72	male	urinary frequency/dysuria/painful urination	bladder/urethra	Cystoscopy biopsy/None	Small	No description	Dead	62
9	72	male	urinary frequency/painful urination	bladder	Radical cystectomy	Mixed	Yes	Survival	7
10	65	female	hydronephrosis	ureter	Nephroureterectomy+Partial cystectomy	Small	Yes	Survival	2

None for pathological type: diagnosed as neuroendocrine neoplasm without further subtype classification.

No description for muscularis propria invasion: muscularis layer was inadequately sampled or incomplete, so not assessed.

### 3.2 Surgical management

Of the 10 patients, 2 underwent transurethral resection of bladder tumor, 2 underwent radical cystectomy with cutaneous terminal ureterostomy, 1 underwent radical cystectomy, 1 underwent partial cystectomy, 1 only received cystoscopic biopsy, and 2 underwent unilateral nephro-ureterectomy with bladder sleeve resection (one underwent para-aortic lymph node dissection, while the other underwent unilateral nephro-ureter-adrenalectomy combined with retroperitoneal lymph node dissection).

### 3.3 Pathology

According to the pathological findings, 4 cases were diagnosed as mixed neuroendocrine-non-neuroendocrine carcinoma, 2 as large cell neuroendocrine carcinoma, 2 as small cell neuroendocrine carcinoma, and 2 were not otherwise specified. Regarding muscular layer invasion, 6 cases showed muscular layer invasion, 1 case showed no muscular layer invasion, and the remaining 3 cases were not documented.

### 3.4 Survival outcomes

Regarding outcome events, 3 patients died, all of whom had bladder lesions. Among them, one underwent partial cystectomy, one underwent radical cystectomy, and one was diagnosed only by cystoscopy. None of these three patients received radiotherapy or chemotherapy. Among the 6 surviving patients, 5 received postoperative radiotherapy or chemotherapy, and 1 underwent regular follow-up alone. One patient was lost to follow-up. For urinary tract neuroendocrine neoplasms (UT-NENs), postoperative platinum-based chemotherapy and radiotherapy tended to have more favorable survival outcomes.

## 4 Discussion

Neuroendocrine neoplasms (NENs) can be classified in various ways (4). Based on their origin, NENs can be divided into primary and metastatic tumors. Primary urinary tract NENs are rare, accounting for less than 1% of urothelial tumors (2). According to the 2016 World Health Organization classification, NENs include highly clinically aggressive tumors (small cell neuroendocrine carcinoma and large cell neuroendocrine carcinoma), well-differentiated neuroendocrine tumors, and paragangliomas (3, 5). NENs can also be categorized into simple and mixed types. Studies suggest that mixed NENs are more common than simple-type NENs in the urinary system (6–10). Mixed NENs refer to tumors composed of different neuroendocrine subtypes or a combination of neuroendocrine and non-neuroendocrine components. The most common non-neuroendocrine component is urothelial carcinoma (6, 11, 12).The pathogenesis of ureteral NEN remains unclear, but four hypotheses have been proposed to explain its origin (13):(1) primitive urothelial cancer cells undergo neuroendocrine metaplasia;(2) carcinogenesis arises from the ureteral submucosa or normal urothelium;(3) abnormal migration of neural crest cells during embryonic development leads to their retention in the ureter and subsequent malignant transformation;(4) cancer stem cells undergo neuroendocrine metaplasia. It is widely accepted that NENs originate from undifferentiated stem cells with multipotent differentiation potential (6, 14, 15). In addition, some scholars classify NENs according to their location, pathological histomorphology, or other characteristics.

In the urinary system, the bladder is the most common site for neuroendocrine neoplasms (NENs), whereas ureteral NENs are extremely rare (6). Additionally, the incidence of urinary tract NENs appears to be higher among elderly Asian males than among other ethnic groups (13).NENs exhibit diverse and non-specific clinical manifestations, making differentiation from urothelial neoplasms and metastatic carcinomas challenging. Therefore, early definitive diagnosis relies on pathological examination. The initial symptoms of urinary tract NENs are predominantly back pain and hematuria (16), with other common symptoms including dysuria, frequent urination, and urinary urgency (6). A few cases may present insidiously: asymptomatic patients are often identified during routine health check-ups (17). Imaging studies may demonstrate ureteral dilation, obstruction, stenosis, hydronephrosis, or urinary system masses, among other findings. Renal or ureteral NENs typically occur unilaterally (13); due to compensation by the contralateral healthy kidney, renal function may remain normal in the early phase. Routine serum tests alone may result in missed diagnoses (16), highlighting the crucial role of timely imaging examinations for early detection.

The diagnosis of urinary tract neuroendocrine neoplasms (UT-NENs) primarily relies on pathological morphological features and immunohistochemical (IHC) findings, including neuroendocrine markers such as CD56, chromogranin A (CgA), synaptophysin (Syn), and epithelial markers (e.g., CKpan). Grimelius staining and the identification of neuroendocrine granules facilitate definitive diagnosis (18).IHC markers for NENs fall into two categories: (1) non-specific epithelial markers, such as epithelial membrane antigen (EMA), cytokeratin (CK), and leukocyte common antigen (LCA); and (2) specific neuroendocrine markers, which are more diagnostically significant, including CgA, neuron-specific enolase (NSE), and Syn (19). Some studies have shown that approximately 70%–90% of NENs express CgA, with a diagnostic specificity of 92% and sensitivity of 96%. In contrast, NSE is more sensitive but less specific for the diagnosis of NENs. Due to the rarity of urological neuroendocrine tumors (NENs), there is currently no consensus on the treatment. The widely used options is surgery with postoperative adjuvant therapy. A cisplatin-based postoperative chemotherapy regimen is the preferred adjuvant therapy (5, 13). According to previous literature, certain clinical and pathological features have been reported to be related to oncologic outcomes in patients with UT-NENs, including female sex, pure small cell carcinoma histology, and pathological stage (5, 6, 16, 18, 20–22). When it comes to the mixed NENs, the neuroendocrine component is frequently reported to drive the overall biological behavior and prognosis (7, 8, 23, 24). Therefore, we should pay more attention to the neuroendocrine part of the tumor during treatment. Besides, some researchers have suggested that pathological stage is potentially related to the prognosis of UT-NENs (18).

According to **Table 1**, among the 10 patients in this study, 6 were elderly males, 2 were elderly females, and 2 were middle-aged males. This finding is consistent with previous reports indicating a higher prevalence of NENs in elderly males (13, 18). Of the identified lesions, seven were located in the bladder and three in the ureter, a distribution consistent with bladder NENs being the most prevalent subtype. In our study, 5 of the 6 patients presenting with hematuria or urinary irritation symptoms had bladder lesions. Patients with bladder neoplasms are more likely to seek medical attention due to these overt symptoms, making bladder NENs more easily detected and potentially introducing selection bias. Postoperative pathological results did not suggest an obvious association between these symptoms and muscularis propria invasion.

Among the 3 patients with ureteral lesions, 2 were initially found to have hydronephrosis accompanied by muscularis propria invasion of the ureteral wall. Given that ureteral NENs are often asymptomatic, the presence of hydronephrosis may indicate an advanced disease stage (13).

This case series summarizes the clinical features of 10 urinary system neuroendocrine neoplasms, supplements clinical data for such rare tumors, and helps improve disease recognition and diagnosis. Nevertheless, as a retrospective case series with small sample size, it carries potential selection bias, and the observations require further validation in larger cohorts.

## 5 Conclusion

Against the background of scarce real-world clinical data of UT-NENs in the Asian population and insufficient clinical awareness of this rare tumor, this report enriched the clinical database of UT-NENs and filled the relevant knowledge gap in clinical practice. In recent years, the incidence and prevalence of urinary tract neuroendocrine neoplasms (UT-NENs) have been steadily increasing (5). In clinical practice, a comprehensive evaluation—particularly preoperatively—is of great significance for improving patient prognosis (25). With advances in medical and medical device technology, the management of UT-NENs has advanced significantly. Patients with UT-NENs who receive multimodal therapy may show a trend toward better overall survival than those who undergo single-modality treatment alone. Preoperative biopsy to determine the pathological type enables clinicians to more accurately assess patients’ conditions and develop more targeted clinical strategies, which may exert a positive effect on both treatment efficacy and prognosis.

## Data Availability

The original contributions presented in the study are included in the article/supplementary material. Further inquiries can be directed to the corresponding authors.
